# Health Risks Due to Metal Concentrations in Soil and Vegetables from the Six Municipalities of the Island Province in the Philippines

**DOI:** 10.3390/ijerph19031587

**Published:** 2022-01-30

**Authors:** Ronnel C. Nolos, Christine Joy M. Agarin, Maria Ysabel R. Domino, Pauline B. Bonifacio, Eduardo B. Chan, Doreen R. Mascareñas, Delia B. Senoro

**Affiliations:** 1 Mapua-MSC Joint Research Laboratory, Marinduque State College, Boac 4900, Philippines; rcnolos@mapua.edu.ph (R.C.N.); mydomino@mapua.edu.ph (M.Y.R.D.); pauline.bonifacio07@gmail.com (P.B.B.); 2 Department of Environmental Science, College of Natural and Allied Health Sciences, Marinduque State College, Boac 4900, Philippines; 3 Resiliency and Sustainable Development Center, Yuchengco Innovation Center, Mapua University, 658 Muralla St., Intramuros, Manila 1002, Philippines; 4 Analytical Support Services for Environmental Technologies, Incorporated, Clark Freeport Zone, Angeles City 2009, Philippines; christinejoyagarin@gmail.com; 5 Dyson College of Arts and Science, Pace University, New York, NY 10038, USA; echan@pace.edu; 6 School of Agriculture, Fisheries and Natural Science, Marinduque State College, Torrijos 4903, Philippines; doreen.mascarenas@g.msuiit.edu.ph; 7 School of Civil, Environmental and Geological Engineering, Mapua University, 658 Muralla St., Intramuros, Manila 1002, Philippines

**Keywords:** vegetables, soil, metals, health risks, pollution, risk assessment, island

## Abstract

This paper investigated the health risks due to metal concentrations in soil and vegetables from the island province in the Philippines and the potential ecological risks. The concentrations of Cd, Cr, Cu, Fe, Mn, Ni, Pb, and Zn in vegetables and soil in six municipalities of the province were analyzed using the Inductively Coupled Plasma Optical Emission Spectrometry (ICP-OES) Perkin Elmer Optima 8000. It was recorded that all metal concentrations in the soil, except for Cd, exceeded the soil quality standard (SQS). The concentration of Fe and Mn was highest among other metals. The Nemerow synthetical pollution index (P_n_) in all soil samples was under Class V which means severe pollution level. Likewise, the risk index (RI) of soil ranged from high to very high pollution risk. Most of the metal concentrations in the vegetables analyzed also exceeded the maximum permissible limit (MPL). All health hazard indices (HHIs) were less than 1, which means potential low non-carcinogenic risk to human population by vegetable consumption. However, it was found that concentration of Cr and Ni in vegetables is a potential health hazard having concentrations exceeding the maximum threshold limit. A 75% temporary consumption reduction of bitter melon, eggplant, sweet potato tops, and string beans produced from two municipalities may be helpful in reducing exposure to target metals. Additional studies are needed to confirm this recommendation. Spatial correlation analysis showed that six out of target metals had datasets that were more spatially clustered than would be expected. The recorded data are useful for creation of research direction, and aid in developing strategies for remediation, tools, and programs for improving environmental and vegetable quality monitoring.

## 1. Introduction

The island province of Marinduque in the Philippines, with farming as the major source of income of the population, had been recognized as one of the country’s largest copper reserves. Hence, copper mining operations were carried out in Marinduque since 1969 [1]. In 1964, the mining industry was established in the province [2]; however, the Maguilaguila siltation dam, a tailing pond, collapsed on 6 December 1993, allowing mine tailings to flow into the Mogpog River [3]. Mine tailings swamped the barangays along the river. On 24 March 1996, almost three years after the Maguilaguila mining disaster, the Tapian dam fell, flooding the 27-km-long Boac River with mine tailings [3]. The Boac River tailings deposits were thought to be a long-term source of acid and elevated concentrations of metals in the environment. Significant amounts of soluble salts have built up due to oxidation of sulfides in the tailings [4]. These salts retain acids and metals in a solid state that is easily soluble until the next rainfall. The cycle of salt formation and dissolution can be repeated each dry and wet season [5]. Currently, the two open mine pits, i.e., Maguilaguila and Tapian pits, that were abandoned when mining operation stopped in 1997 still exist in Marinduque and these are located at a higher elevation in the municipality of Sta. Cruz. Therefore, there is a serious concern that mine tailings will contaminate the biomass of plants and animals in the environment [6] and exacerbate health concerns [7]. 

Toxic metals have been related to a number of health problems [8]. Some of these metals are lead (Pb), nickel (Ni), arsenic (As), cadmium (Cd), and chromium (Cr) that have been classified as human carcinogens (known or probable) by the US Environmental Protection Agency (USEPA) [9] and the International Agency for Research on Cancer (IARC) [10]. Another condition that adds to the public concern is the nature of the geological profile of the island province of Marinduque. It is composed of volcanic and/or sedimentary rocks that are porous and can transmit contaminants from higher to lower elevations [11]. Environmental contamination has the capacity to migrate across land surface and underground porous media [12]. Due to the essential characteristics of heavy metals such as accumulation [13], bioavailability [14], and low mobility in soils [15], heavy metal pollution poses both potential ecological and health risks [16]. Hence, the quality of vegetables and edible crops produced in the island province has become a public concern [17]. Vegetables are an important dish and most often part of regular meals for the Philippine population and the global population. It has been noted that vegetables are an important dietary component for humans. Some vegetables contain metals and are important micronutrients for living organisms. Hence, giving attention to the quality of vegetables and soil is important.

Plants absorb metals from the soil and water through the roots, hence, the presence of elevated metal concentrations in these media affects the quality of plants [11]. Crops and vegetables grown in contaminated soils can accumulate significant levels of hazardous metals, posing serious health concerns to humans if consumed [18]. The most common pathway for human exposure to hazardous metals is through food consumption. Heavy metals are easily absorbed by vegetable roots and can build up to large levels in the edible sections of vegetables, even at low levels in the soil [19]. Moreover, low-level chronic exposure to heavy metals can have long-term health consequences [20].

There have been several studies on heavy metal contamination in water and sediment [21,22,23,24] in the island province; however, metal concentrations in soil and vegetables were not examined. Other studies focused on phytoremediation strategies and techniques [25,26,27,28,29]. These studies recorded the highest concentration of Zn in the roots, Cu in the stems, and Pb in the leaves [29]. Additionally, Cu accumulated in the roots more than twice the rate of other parts of the plants [30]. Moreover, heavy metal contaminations are frequently compounded with numerous metals in the field [31]. Hence, more research on the quality of various vegetables grown and consumed in the province is needed. These studies are possible toxicity [32], resistant nature [33], speciation [34], frequency of vegetable intake, safety and health issues [35], as well as uncertainty and variability analysis [36,37,38]. Most health risk assessments are made up of primary, secondary, processed data, and engineered models. These engineered models are under conditions of probability considering the significant contribution of relative uncertainty and variability [36,37,38]. Hence, health risk assessment approaches incorporated the correlation and statistical analysis techniques to address uncertainties and variability. In this paper, the levels and trends of metal concentrations in soil and vegetables from the island province in the Philippines, the potential health risks associated with their daily use, and how the soil and vegetables are spatially correlated are presented. 

## 2. Materials and Methods

### 2.1. Study Area and Collection of Samples

Marinduque is an island province, with about 53,344 households in the Republic of the Philippines with coordinates of 13.4767° N, 121.9032° E. This island province has a tropical climate with annual mean temperature of 27 °C. The island does not have a distinct wet and dry season as precipitation occurs all year. 

**Figure 1:**
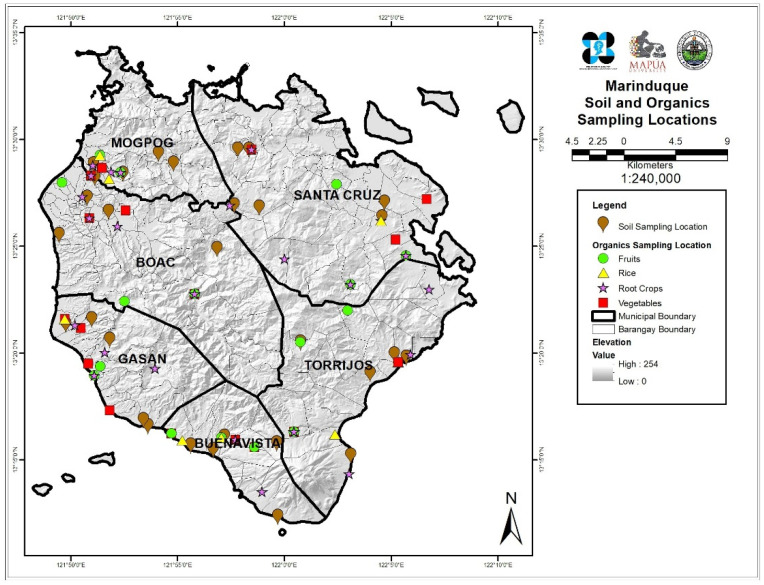
Map of the study area and locations of sampling sites.

The vegetable and soil samples were collected from six municipalities of Marinduque, namely Boac (B), Buenavista (BV), Gasan (G), Mogpog (M), Sta. Cruz (S), and Torrijos (T). Sampling locations were distributed spatially over the six municipalities of the island province. Coordinates of every sampling point were recorded and plotted using Geographic Information Systems as shown in Figure 1. Generally, the land use of the sampling points from the island province is mainly agricultural [39,40].

Four types of vegetables samples were collected in the individual towns’ public markets and household farmyards. A total of 96 samples were assessed, analyzed, and evaluated in triplicates. The vegetables were chosen from the top produced and consumed vegetables in Marinduque, namely string beans (*Vigna unguiculata*), sweet potato tops (*Ipomoea batatas*), bitter melon (*Momordica charantia*), and eggplant (*Solanum melongena*). Likewise, composite soil samples were collected from each municipality across the province with a stainless-steel auger at 0–30 cm depths. The selection of sites was undertaken on the basis of where vegetables farms are located. Coordinates were recorded using a handheld Garmin global positioning system (GPS) model Montana 650. All vegetable and soil samples were put in a zipper bag, labeled, and transported to the laboratory for treatment and analysis. Sample collection started in 2018 and ended in 2019 completing one year season.

### 2.2. Samples Preparation and Analysis

Vegetable samples were washed, thinly cut/sliced, and dried in a dehydrator at 68 °C for 7–8 h at the MSC-Mapua joint research laboratory in Boac, Marinduque. Dried samples were pulverized into fine particles and stored at room temperature in a properly labeled disposable Petri dish until digestion. Utensils and laboratory equipment were cleaned with soap and water, then rinsed with 1.5% *v*/*v* nitric acid and distilled water every use per sample. Additionally, the soil samples were dried at 68 °C for 3 h, ground to a fine powder using an agate mortar and pestle, homogenized, and passed through a 10-mesh sieve before analysis. The concentration of metals such as cadmium (Cd), chromium (Cr), copper (Cu), iron (Fe), lead (Pb), manganese (Mn), nickel (Ni), and zinc (Zn) was analyzed in triplicates using an Inductively Coupled Plasma Optical Emission Spectrometer (ICP-OES) Perkin Elmer Optima 8000. The ICP-OES used in this analysis has detection limits of 0.05, 0.1, 0.1, 0.5, 0.07, 0.5, 0.1, 0.04, and 2.0 for Cd, Cr, Cu, Fe, Hg, Mn, Ni, Pb, and Zn, respectively. The EPA Method 3050B [41], 200.3 [42], and 6010c [43] were followed in the digestion and analysis of samples. The digestion and analysis were conducted at Mapua University’s Yuchengco Innovation Center’s Wet Laboratory in Manila, Philippines.

### 2.3. Evaluation of Metal Pollution in Soil

The level of soil pollution was assessed by the single-factor (*P_i_*) and the Nemerow synthetical pollution index ($P_{n}$) [44]. The *P_i_* is a useful tool for determining the pollution degree of a certain heavy metal in soil using Equation (1).

(1) $$P_{i}=\sqrt{\left(\frac{C_{i}}{S_{i}}\right)}$$

where $C_{i}$ is the concentration of metal *i*, and $S_{i}$ is the soil quality standard value [45,46]. A higher value of $P_{i}$ indicates more serious pollution, and soil is considered as polluted when $P_{i}$ is greater than 1 (Table 1). 

To better describe the level of soil pollution when *P_i_* is less than 1, the $P_{n}$ can evaluate a range of heavy metal toxicity levels, as shown in Equation (2).

(2) $$P_{n}=\sqrt{\frac{\left(P_{imax}^{2}+P_{iave}^{2}\right)}{2}}$$

where $P_{imax}$ is the maximum value of the *P_i_* and $P_{iave}$ is the average value of the *P_i_*. The grading standard for *P_i_* is shown in Table 1.

### 2.4. Potential Ecological Risk Index (pERI) 

To measure the adverse effects of soil contamination, the risk index (RI) approach established by Hakanson [47] was used. The approach requires four hypotheses elaborated by the work of Agarin et al. [3]. The potential ecological risk index (pERI) considers the concentrations of metals, their environmental impact, and biotoxicity. The RI is computed by following Equation (3).

(3) $$RI=\overset{n}{\underset{i=1}{\sum }}P_{i}\times T_{i}$$

where $T_{i}$ is the toxic response factor for metal *i* [44]. The grading standard for pERI is also categorized in Table 1.

### 2.5. Potential Human Health Risk Assessment

The potential human health risk from the consumption of vegetables was estimated by calculating the estimated daily intake (EDI) of metals, health hazard index (HHI), and target cancer risk (TCR) [3]. The EDI in Equation (4) was used to calculate the level of exposure to a particular metal in vegetables via ingestion/oral route [48].

(4) $$EDI=\frac{E_{f}\times E_{D}\times F_{IR}\times C_{m}\times C_{f}}{B_{w}\times T_{A}}\times 10^{-3}$$

where, $E_{f}$ is exposure frequency (365 days/year); $E_{D}$ is the exposure duration (years); $C_{m}$ is the metal concentration (mg kg^−1^); $F_{IR}$ is the daily average vegetable consumption (g person^−1^ day^−1^) [49,50]; $C_{f}$ is the conversion factor for fresh vegetable weight to dry weight (0.085) [51,52]; $B_{w}$ is the reference body weight for an adult (kg); and $T_{A}$ is the average exposure time. The exposure duration considered was 70 years [53,54,55]. This is based on the average life expectancy of the Philippine population [53,54]. The Philippine population $F_{IR}$ for bitter melon, eggplant, string beans, and sweet potato tops are 7.42, 14, 6.99, and 7.78 g person^−1^ day^−1^ [49,50], respectively.

The target hazard quotient (*THQ*) provides an estimate of the amount of risk associated with pollutant exposure [56]. It was used to evaluate the potential health risks of metal consumption through vegetables [19]. Equation (5) was used to estimate the THQ values of the population as a result of consuming contaminated vegetables as described by Chen et al. [57] and Ezemonye et al. [58].

(5) $$THQ=\frac{EDI}{R_{f}D}$$

where, $R_{f}D$ is the oral reference dose (mg kg^−1^ day^−1^) [56,59]. The *R_f_D* is an estimate with uncertainty spanning an order of magnitude of a daily oral exposure to the human population that is likely to be without an appreciable risk of deleterious effects during a lifetime [60]. This number was based on the no-observed adverse effect level (NOAEL) with the consideration of uncertainty factor. If the estimated daily intake of contaminated vegetables of a population is greater than the *R_f_D*, then the occurrence of associated health risks increases. Should the *THQ* be less than one, it is considered safe or has non-carcinogenic consequences. Should *THQ* be larger than one, the probability of danger increases [19,56].

The *HHI* [3] was determined by adding the individual metal target hazard quotient values [61] using Equation (6).

(6) $$HHI=\overset{n}{\underset{i=1}{\sum }}THQ_{i}$$

Carcinogenic risk assessment, on the other hand, is an estimation of the cumulative probability of developing cancer throughout a lifetime by exposure to a unit dose of a probable carcinogen [34]. The possibility of cancer risks in the studied vegetables through intake of carcinogenic heavy metals was estimated using the cancer risk (CR) [62] as shown in Equation (7). Then, the *TCR* from heavy metals intake, which can have a carcinogenic effect depending on the exposure level [63], was calculated using Equation (8).

(7) CR=EDI×CSF

(8) $$TCR=\overset{n}{\underset{i=1}{\sum }}{CR}_{i}$$

where, *CSF* is the cancer slope factor and defined as the risk generated by a lifetime average amount of 1 mg kg^−1^ day^−1^ of carcinogen chemical and is contaminant specific [63,64,65,66]. Therefore, cancer risk was expressed in terms of incremental lifetime cancer risk, i.e., the probability of developing cancer over a 70-year lifetime period due to a 24 h exposure to a potential carcinogen [55]. The values of *CSF* for Cd, Cr, Ni, and Pb are 0.38, 0.5, 1.7, and 0.0085 mg kg^−1^ day^−1^ [63], respectively. In contrast, if the *TCR* exceeds the maximum threshold value of $1.00\times 10^{-4},$ there is a high risk of carcinogen exposure [63,65,66,67].

### 2.6. Spatial Correlation Analysis

ArcGis Desktop 10.8.1 ArcPro 2.8 [68] was employed to create spatial distribution maps and spatial correlation analysis. Moran’s I (index) was used to determine the autocorrelation of datasets to produce spatial distribution maps of metal concentrations in soil and vegetables. Moran’s I is classified as positive, negative, and no-spatial autocorrelation. Positive autocorrelation occurs when values clustered together or appear near to each other. Negative spatial autocorrelation occurs when datasets are dispersed, i.e., dissimilar values occur next or near to each other. Should similar and dissimilar values appear randomly, then no spatial autocorrelation occurred.

### 2.7. Statistical Analysis

Pearson’s correlation and hierarchical cluster analysis (HCA) was used using IBM SPSS Statistics Version 26.0 to look at the specific relationships between the various metals found in soil and vegetables as well as how certain metals influence their concentration.

## 3. Results and Discussion

Subsequent sections elaborate and discuss the results and the potential health implications of recorded concentrations of metals. Data presented below are helpful in determining research directions, creating strategies in soil remediation, soil quality monitoring programs for vegetable quality monitoring, creation of in situ detection tools for metals specific for vegetables and crops, and among other related techniques helpful for reducing health risks. 

### 3.1. Concentration of Metals in Soil

The soil samples recorded concentrations of Cr (854–2465.86), Cu (1711.27–17,712.23), Fe (759,560.17–1,083,607.03), Mn (25,596.87–60,549.73), Ni (536.43–3216.47), Pb (393.27–1291.13), and Zn (2291.37–6160.83) mg kg^−1^. All the metal concentrations in the soil, except for Cd, from the six municipalities exceeded the soil quality standards (SQS). There are very limited related studies in the area; however, results are comparable to the work of Marges et al. [7], Senoro et al. [69], and Sanchez et al. [70]. The work of Marges et al. and Sanchez et al. in 2009 and 2015 at the Calancan Bay of Marinduque published in March 2011 and January 2018, respectively, showed no recorded Cd concentration in soil. The non-detection of Cd in soil in this study has been attributed to the soil acidity and uptake of some plants [71]. The study of Usman [71] specifically mentioned that plants remediate Cd by phytoextraction and it accumulates in the shoot. Phytoremediation activities in Marinduque were recorded during the period of 2006–2017 [25,26,27,28,29,30]. Other studies in Vietnam [72], Malaysia [73], and Indonesia [74] where there were mining activities recorded an elevated concentration of Cr in soil with no record of Cd concentration. 

It shows that Fe and Mn had the highest concentration of metals in soil across the six municipalities. The high concentration of Fe in the soil can be attributed to the mine tailings from the previous milling of sulfide ores, i.e., copper and zinc, which may have high levels of pyrite (iron sulfide) [75,76]. Likewise, continuous subsurface flow of metals within the island are associated to the two abandoned open mine pits that are located at the higher elevation of the island province [3]. Metal concentration varies spatially as illustrated in Appendix A. Table 2 shows the mean concentrations of metals in the soil samples highlighting the SQS [45,77]. The trend of metal concentrations across the island province is shown in Table 3.

### 3.2. Evaluation of Metal Pollution in Soil 

**Figure 2:**
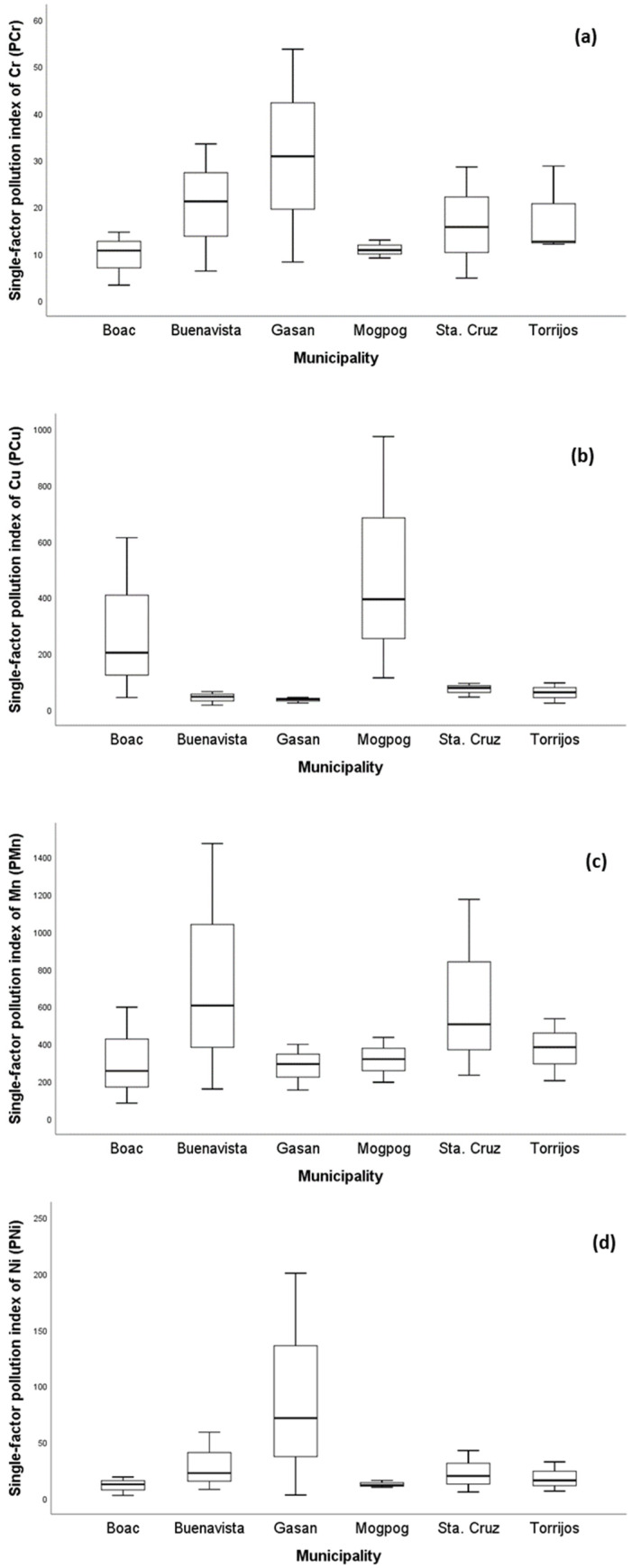

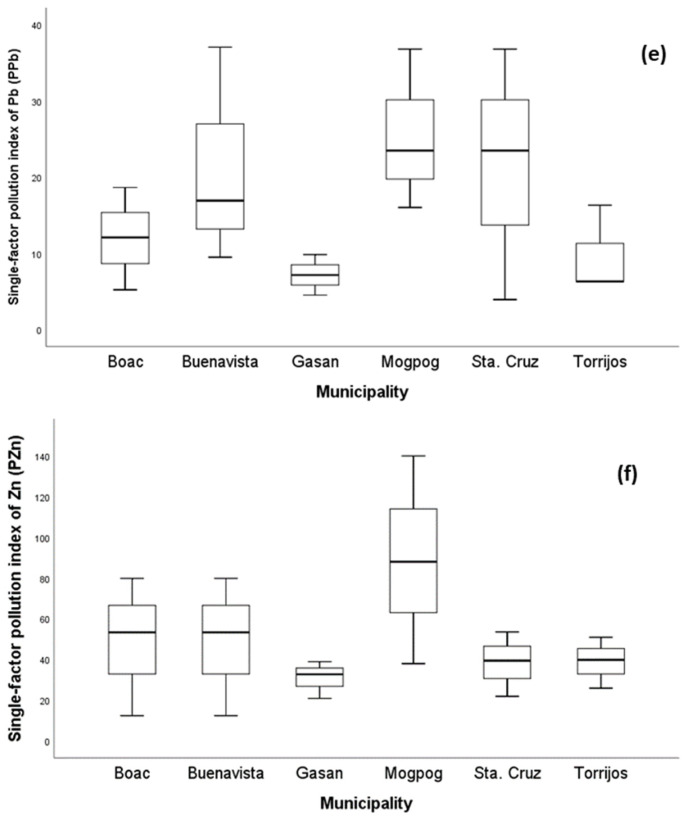
The single-factor pollution index ($P_{i}$) of soil per municipality of the island province. The $P_{i}$ of (**a**) Cr, (**b**) Cu, (**c**) Mn, (**d**) Ni, (**e**) Pb, and (**f**) Zn in soil from all municipalities across the province.

In order to obtain the status of soil pollution from each municipality, the values of $P_{i}$ and $P_{n}$ were calculated using the Nemerow pollution index (NPI). The NPI illustrates the degree of probable pollution a metal contributes, and/or how several metals probably pollute a target environmental medium. As illustrated in Figure 2, all mean $P_{i}$ values of soil from each municipality were greater than five except for Cd which was not detected. This implies that the soil from all municipalities belongs to Class V with severe pollution level (Table 1) and is quite alarming [78]. Heavy metal pollution of soil is a serious environmental hazard, particularly in locations where soils intended for agricultural practices are adjacent to sources of pollution such as mining [44].

**Figure 3:**
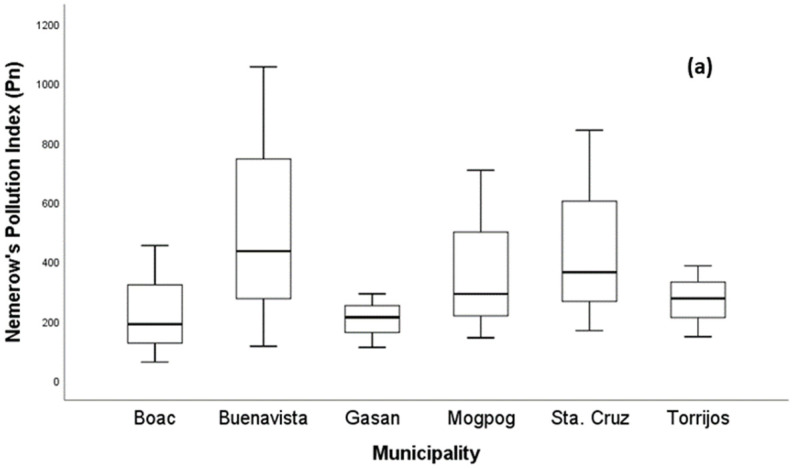

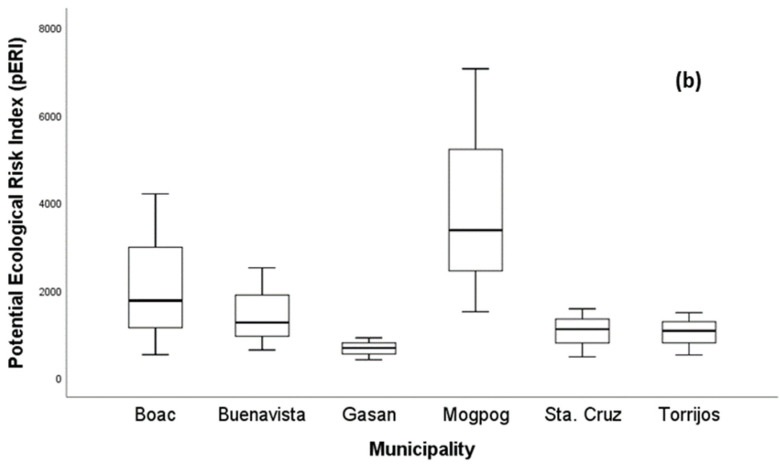
Boxplot of (**a**) the Nemerow synthetical pollution index and (**b**) risk index of metal in soil.

As illustrated in Figure 3a, the $P_{n}$ values in all soil from each municipality ranged from 61.35 to 1055.83, which are greater than three indicating that all soil samples were under Class V with severe pollution level (Table 1). The $P_{n}$ of soil in all municipalities had the following order: BV > S > M > T > G > B. In Figure 3b, the higher pERI was found in the soil of Boac and Mogpog in comparison with Buenavista, Gasan, Sta. Cruz, and Torrijos. More specifically, in Mogpog, the single ecological risk index ($E_{r}^{i}$) of Cu was greater than 1200, ($E_{r}^{i}$ = 1968.03), indicating a higher contribution of Cu to RI. The RI of soil in Boac, Buenavista, and Mogpog exceeded 1200; hence, there is very high pollution risk. On the other hand, the RIs of Gasan, Sta. Cruz, and Torrijos were between 600 and 1200 indicating a high pollution risk too. The high pollution risk of the soil due to metal pollution can adversely impact plant growth [79], microbial diversity [80], biota [81], and humans whose exposure includes incidental ingestion, inhalation, and dermal contact [82].

### 3.3. Concentration of Metals in Vegetables

Firstly, it must be emphasized that metals such as Fe and Zn are essential micronutrients for human health and other living organisms. The degree of concentrations of metals in vegetables presented below are useful in making strategies, epidemiological studies, further research on edible agricultural yields, and creating more tools for vegetable quality monitoring to protect human health. It will also aid comprehensive soil and vegetable quality monitoring.

**Figure 4:**
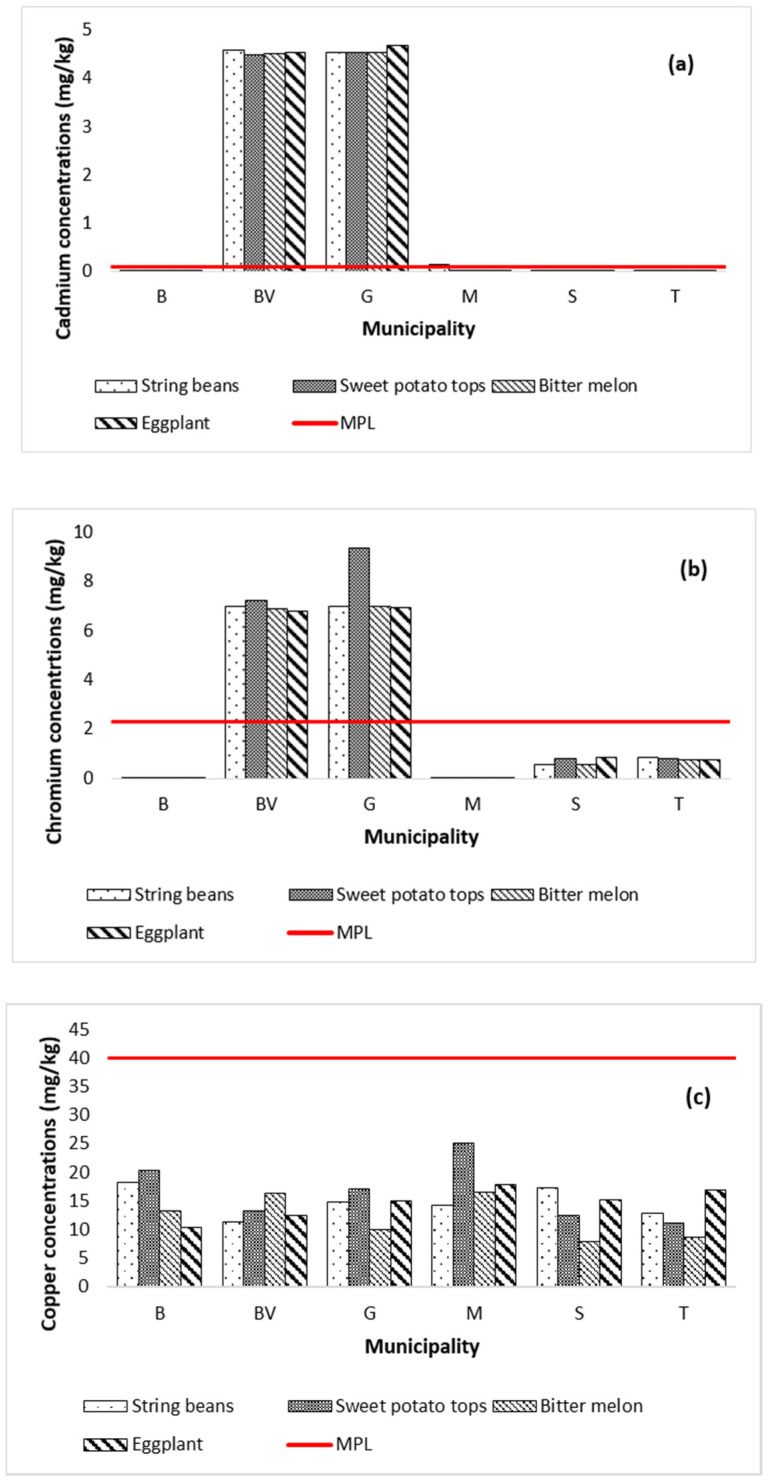

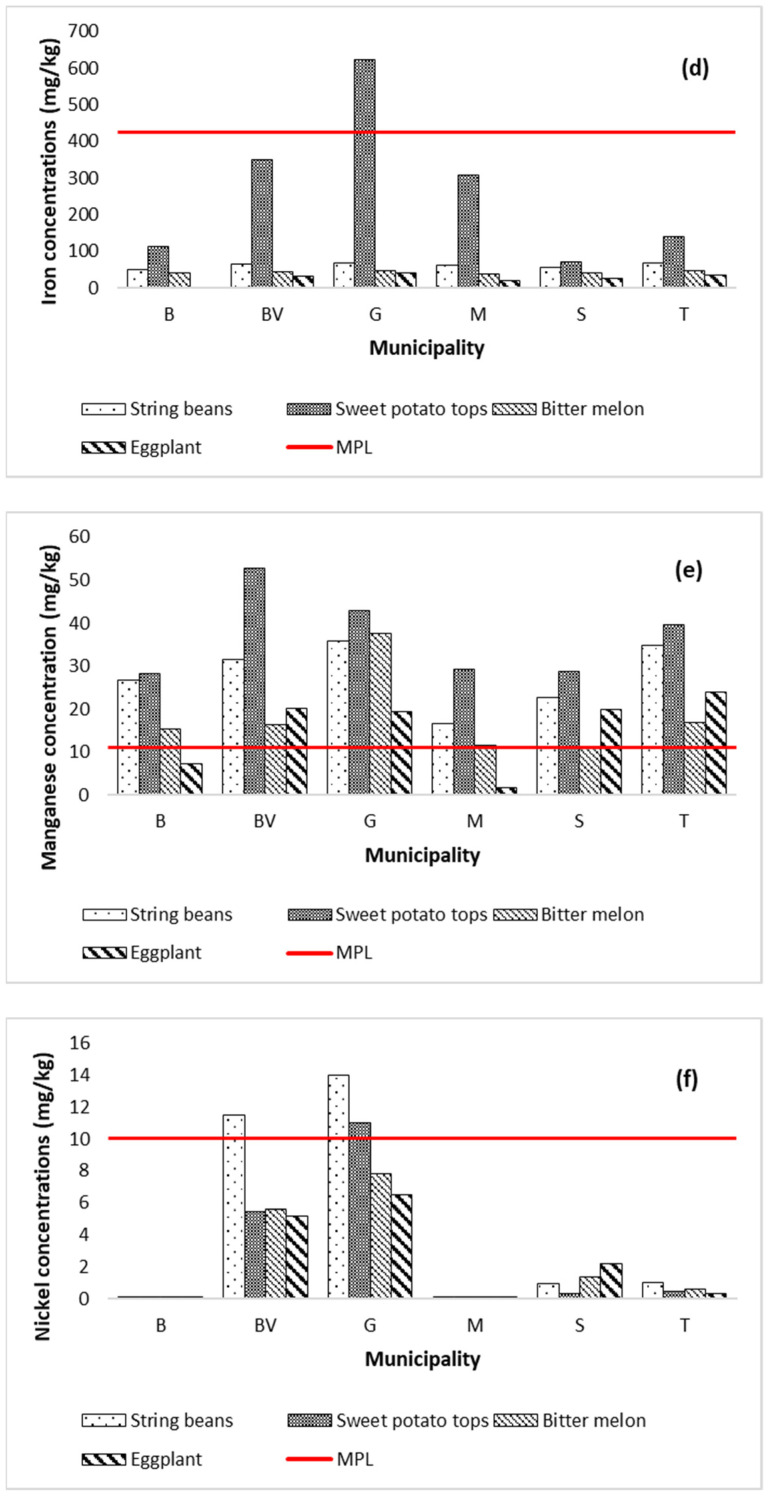

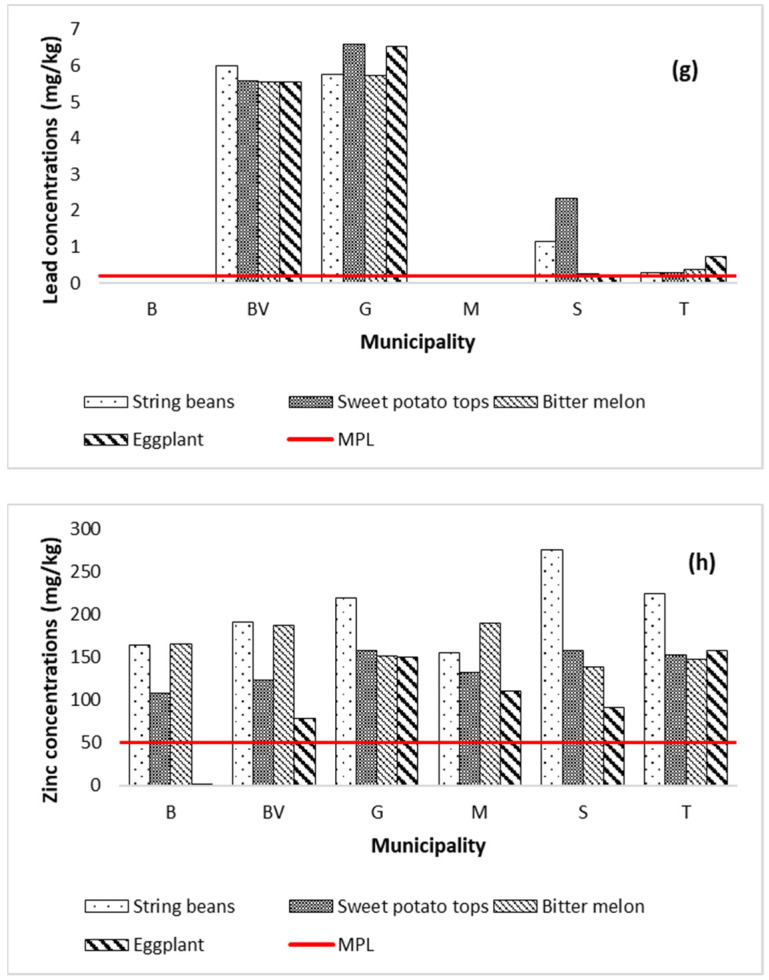
Metal concentrations in vegetables by municipality in the island province. The concentration of Cd (**a**) in all vegetables from BV and G was above MPL. The concentration of Cr (**b**) in vegetables from BV and G was above the MPL. The Cu concentration (**c**) in all vegetables was below MPL in all six municipalities. The Fe concentration (**d**) in all vegetables of the six municipalities except for the sweet potato tops of G was below MPL. The Mn concentration (**e**) in all vegetables from the six municipalities except the eggplant from B and M was beyond MPL. The Ni concentration (**f**) in string beans from BV and G and the sweet potato tops from Gasan exceeded the MPL. The Pb concentration (**g**) in all vegetables from BV, G, S, and T was beyond MPL. The Zn concentration (**h**) in all vegetables from the six municipalities except for the eggplant of B was above the MPL.

The mean concentrations (mg kg^−1^) of metals in the vegetable samples collected from Marinduque are shown in Figure 4a–h with a red horizontal line representing the maximum permissible limits (MPL) set by World Health Organization, Food and Agriculture Organization and International Food Standards [82]. Metal concentrations in vegetables are represented by Y-coordinates and illustrated by vertical bars. The MPL (mg kg^−1^) for Cd, Cr, Cu, Fe, Mn, Ni, Pb and Zn concentrations illustrated in Figure 4a–h are 0.1, 2.3, 40, 425, 11, 10, 0.2 and 50, respectively [63,67,82,83,84,85]. The string bean samples had concentrations (mg kg^−1^) of Cd (0.004–4.581), Cr (0.0005–6.968), Cu (48.34–68.50), Fe (48.40–68.58), Mn (16.72–35.75), Ni (0.001–13.978), Pb (0.012–5.986), and Zn (155.98–275.50). Metal concentrations in sweet potato tops ranged from 0.004 to 4.531 Cd, 0.0005–9.323 Cr, 11.13–24.99 Cu, 70.84–625.35 Fe, 28.21–52.84 Mn, 0.001–11.022 Ni, 0.012–6.581 Pb, and 107.73–157.99 Zn. The bitter melon samples had concentrations of Cd (0.004–4.541), Cr (0.0005–6.9625), Cu (7.95–16.44), Fe (37.70–47.76), Mn (11.26–37.54), Ni (0.001–7.811), Pb (0.01–5.74), and Zn (138.54–190.24). Metal concentrations in eggplant range from 0.004 to 4.675 Cd, 0.0005–6.9128 Cr, 10.433–17.822 Cu, 3.20–39.57 Fe, 1.86–23.94 Mn, 0.001–6.494 Ni, 0.012–6.533 Pb, and 0.001–157.630 Zn. It was recorded that Zn concentration in all types of vegetables across the island province exceeded the MPL except for the eggplant collected from Boac. The Fe in sweet potato tops collected from Gasan municipality exceeded the MPL. Additionally, the concentration of Mn in all types of vegetables, except for the eggplant collected from Mogpog, exceeded the MPL. Likewise, Cd and Cr concentrations in most types of vegetables from Buenavista and Gasan exceeded the MPL. It was also recorded that concentration of Pb in most types of vegetables from Buenavista, Gasan, and Sta. Cruz exceeded the MPL. Only the string beans from Buenavista and Gasan and the sweet potato tops from Gasan exceeded the MPL for Ni. The spatial distribution map of metal concentrations in soil and vegetables is illustrated in Appendix A to further visualize the potential distribution of metal concentrations across the island province.

It is useful to note that the leafy vegetable (sweet potato tops) had accumulated the highest metal concentrations. Similar findings have been reported by Luo et al. [86], indicating that leafy vegetables accumulate metals more than non-leafy vegetables. The distribution trend of metal concentration in vegetables is shown in Table 4. It shows that Zn had the highest concentration, among target metals in all vegetables across the six municipalities.

### 3.4. Potential Human Health Risk of Metals by Ingestion

**Figure 5:**
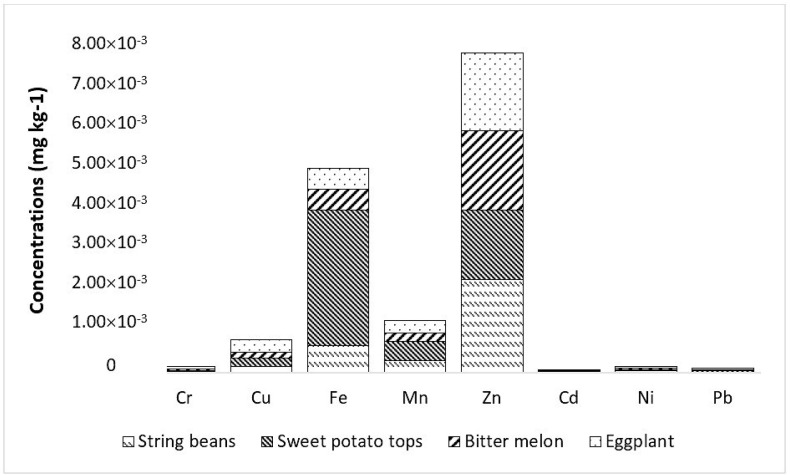
Contribution of vegetables to the average estimated daily intake (EDI) of metals.

**Figure 6:**
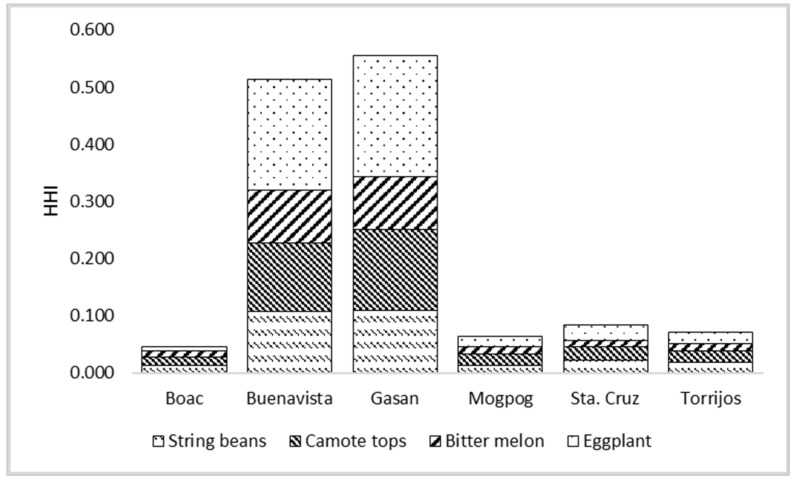
Potential health hazard index (HHI) by ingestion of vegetables from Marinduque.

The EDI of metals through consumption/oral intake of vegetables by the population in Marinduque is presented in Table 5. The eggplant contributed most to the population EDI given that it has the highest $F_{IR}$ among the vegetables tested. It was followed by sweet potato tops then string beans and lastly bitter melon as illustrated in Figure 5. The metals of concern in vegetables were Cd, Cr, Fe, Mn, Pb, Zn, and Cu. The EDI of individual metals as a result of vegetable consumption was in the order of Zn > Fe > Mn > Cu > Ni > Cr > Pb > Cd. Bar plots are illustrated in Figure 5 and Figure 6 to visualize the concentration of metals in each vegetable type and the potential contribution of specific vegetables to the HHI of each municipality.

The TCR associated with exposure to heavy metals such as Cr, Cd, Ni, and Pb from vegetable consumption was calculated using EDI data (Table 5), CSF [63,78,79], and TCR data shown in Table 6.

The computed EDI ranges for various vegetables were $3.85\times 10^{-8}$–$9.86\times 10^{-5}$ Cd, $5.67\times 10^{-9}$–$1.46\times 10^{-4}$ Cr, $6.76\times 10^{-5}$–$7.31\times 10^{-3}$ Fe, $3.92\times 10^{-5}$–$6.20\times 10^{-4}$ Mn, $6.00\times 10^{-9}$–$1.47\times 10^{-4}$ Ni, $1.28\times 10^{-7}$–$1.38\times 10^{-4}$ Pb, $1.85\times 10^{-8}$–$3.16\times 10^{-3}$ Zn, and $8.89\times 10^{-5}$–$3.76\times 10^{-4}$ Cu.

The THQ and HHI values were used to interpret the metals’ possible health hazards. The computed potential HHI of metals present in vegetables is shown in Table 7 which evaluated the cumulative effect of ingesting a variety of potentially harmful metals from several vegetables [63]. Ingestion of the vegetable samples recorded to have THQ values ranged from $3.85\times 10^{-5}$ to $9.86\times 10^{-2}$ Cd, $1.89\times 10^{-6}$–$4.86\times 10^{-2}$ Cr, $9.65\times 10^{-5}$–$1.04\times 10^{-2}$ Fe, $2.80\times 10^{-4}$–$4.43\times 10^{-3}$ Mn, $3.00\times 10^{-7}$–$7.36\times 10^{-3}$ Ni, $1.44\times 10^{-7}$–$3.45\times 10^{-2}$ Pb, $6.18\times 10^{-8}$–$1.05\times 10^{-2}$ Zn, and $2.22\times 10^{-3}$–$9.40\times 10^{-3}$ Cu. All of the HHI values were less than 1, hence, there is a potential low non-carcinogenic human health risk to the human population from vegetable consumption [87]. Further, it was found that the highest HHI values in vegetables were in Buenavista and Gasan. It is being emphasized that THQ and HHI were significantly affected by the EDI and R_f_D values. These values are estimated values with uncertainty.

It can be seen from Table 6 that the TCR of Ni due to vegetable consumption in Buenavista ($6.08\times 10^{-4}$), Gasan ($8.51\times 10^{-4}$), and Sta. Cruz ($1.26\times 10^{-4}$) exceeded the maximum threshold value of $1.00\times 10^{-4}$ [66,67,83,84]. This indicates potential risk of developing cancer if the population has a daily intake of vegetables shown in Table 5. The corresponding TCR of Cr in Buenavista ($1.89\times 10^{-4}$) and Gasan ($2.03\times 10^{-4}$) is also higher than the maximum threshold value. It was observed that ingestion of Cr and Ni in vegetables may pose a cancer risk to the population of Marinduque. This recorded result is similar to the findings of Gebeyehu et al. [63]. Chronic exposure to high amounts of Cr causes malignancies of the gastrointestinal tract, respiratory and central nervous systems [74,76]. However, it is considered that the ingested dose of vegetable with elevated concentration of metals is not always equal to the absorbed metal concentration dose as a fraction could potentially be excreted from the body [88]. Hence, regular excretion is important. Additionally, it is noted that the TCR was based on EDI shown in Table 5, CSF, body weight, and 365 days a year of exposure frequency. Therefore, these variables significantly affect the potential to acquire carcinogens and the likelihood of cancer risk during a lifetime exposure. These elevated metal concentrations in the vegetables that posed HHR to the population were associated to the existence of two abandoned open mine pits which are located at the higher elevation of Marinduque [69]. Groundwater flow [21] through porous media, and runoff overflow in the island province potentially caused the elevated concentration of metals in soil that the vegetables may have absorbed from the soil. Given the above information, it is advised to exercise a 75% temporary consumption reduction of bitter melon, eggplant, string beans, and sweet potato tops produced in the municipalities of Buenavista and Gasan. This is to avoid Cr and Ni TCR. Therefore, the proposed monthly average consumption of bitter melon, eggplant, string beans, and sweet potato tops is 6.9, 7.8, 7.4, and 14 g person^−1^, respectively. This is to reduce exposure and the probability of cancer occurrence by vegetable ingestion. The TCR at 75% reduction is shown in Table 7. The TCR of Ni in Buenavista and Gasan still exceeds the threshold value despite a 75% consumption reduction. Hence, a comprehensive study covering quality of other vegetables produced in the island will be helpful to identify the location and types of vegetables that must be consumed by the public without posing TCR. 

Based on the calculated TCR estimation, a 75% reduction of vegetable consumption would leave Ni alone as concern in the municipalities of Buenavista and Gasan. The TCR of Ni in Buenavista and Gasan at 75% reduction of vegetables consumption is 1.52 × 10^−4^ and 2.13 × 10^−4^, respectively. The recommended 75% consumption reduction is temporary as mentioned above. This recommendation, apart from being temporary, is only for bitter melon, eggplant, string beans, and sweet potato tops produced specifically from the municipalities of Buenavista and Gasan. Hence, the effects of the 75% reduction (referred above) do not violate the “food security (FS)” goal defined by the United Nations’ Committee on World Food Security. The FS definition states that “all people, at all times, have physical social, and economic access to sufficient, safe, and nutritious food that meets their food preferences and dietary needs for an active and healthy life.” The vegetables produced by the above-mentioned municipalities were not safe based on the detected metal concentrations that were beyond the maximum permissible limit [62,66,82,83,84].

### 3.5. Relationships of Metals in Soil and Vegetables

Pearson correlation analysis was carried out to determine the degree of interrelation and association of the metals in soil and vegetables [89]. As shown in Table 8, the correlation coefficients of Ni-Cr, Zn-Pb, and Cu-Zn were 0.917, 0.940, and 0.921 (*p* < 0.01), respectively. The value of (*p* < 0.01) expresses statistical significance of the relationship and association of metals in soil and vegetables.

**Figure 7:**
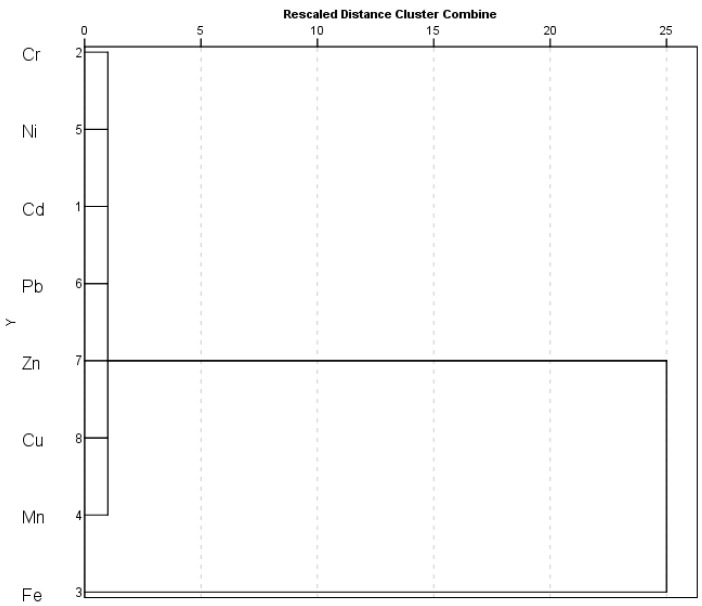
Cluster analysis of metals in soil across the province.

HCA was further used to determine the affinity and behavior of metal in the soil across the province. Figure 7 shows the dendrogram results generated by HCA for metals. The following two primary clusters were identified: (1) Cr-Ni-Cd-Pb-Zn-Cu-Mn and (2) Fe. It produced results that were similar to those of the Pearson correlation analysis.

As shown in Table 9, the correlation coefficients of Cr-Cd, Mn-Fe, Ni-Fe, Ni-Mn, Pb-Cd, and Pb-Cr were 0.986, 0.629, 0.605, 0.852, 0.977, and 0.977 (*p* < 0.01), respectively. These positive significant correlations between these various metals in soil and vegetables demonstrated that they commonly interact, cooperate with each other to promote potential uptake of vegetables from soil, and are a common source of pollution [41,74,75].

Furthermore, spatial correlation technique was employed to analyze further the relationship of metal concentrations in vegetables that were collected in proximity to where the soil samples originated. Table 10 elaborates the generated Moran’s Index that measures the spatial autocorrelation of soil and vegetables. This table illustrates that the concentration of Cr, Ni, Pb, and Zn in the soil is spatially correlated with concentration in vegetables. In addition, though Cd was not detected in soil, the Cd concentration in vegetables shows that it is spatially correlated to nearby environmental media having a Moran’s I and *p*-value of 0.668301 and 0.004269, respectively. This is attributed to the groundwater [21,65] and runoff quality [65].

## 4. Conclusions

In this study, the trends of metals in soil and vegetables across the island province in the Philippines were investigated. Soil and vegetable samples were collected from the six municipalities of Marinduque island province and vegetables chosen were among the top produced and consumed in the province, namely string beans, sweet potato tops, bitter melon, and eggplant. All samples were analyzed for the presence of Cd, Cr, Cu, Fe, Mn, Ni, Pb, and Zn and assessed for potential ecological and human health risks. It was recorded that all metal concentrations in the soil, except for Cd, from the six municipalities exceeded the SQS. The concentrations of Fe and Mn in soil were the highest among other target metals. The *P_n_* values in all soil samples from each municipality were under Class V which means severe pollution level. Likewise, the pERI of soil in Boac, Buenavista, and Mogpog was considered to have a very high pollution risk while for Gasan, Sta. Cruz, and Torrijos, a high pollution risk was found. 

Most of the metal concentrations in the vegetables exceeded the MPL. The sweet potato tops, a leafy vegetable, had accumulated the most metals among the vegetables. Eggplant more likely contributed the greatest TCR to the population. Moreover, all HHI values were less than 1 which means that there is a potential low non-carcinogenic human health risk to the population from the consumption of vegetables. The concentrations of Cr and Ni in target vegetables have the potential risk of cancer occurrence to the population. The TCR of Cr and Ni exceeded the maximum threshold value of 1.00 × 10^−4^. It is further stressed that values of TCR were based on estimated EDI and RfD with uncertainty though addressed by engineering models. Hence, more focused and deeper study is useful. 

Based on this record, it is advised to exercise a 75% temporary consumption reduction of bitter melon, eggplant, string beans, sweet potato tops produced from the municipalities of Buenavista and Gasan. Therefore, the proposed temporary monthly average consumption of bitter melon, eggplant, string beans, and sweet potato tops is 6.9, 7.8, 7.4 and 14 g person^−1^, respectively. However, more studies are necessary to identify more vegetables and from which area they should be produced in order to sustain the dietary and vegetable preference needs of the population.

The datasets were subjected to an autocorrelation analysis using Moran’s Index of the GIS. Records showed that the datasets were more spatially correlated and clustered than would be expected. This means that metal concentrations in soil and nearby vegetables are correlated with each other. Further, the Pearson correlation and hierarchical cluster analyses, on the other hand, revealed that the positive significant correlation between the grouped metals in soil and vegetables demonstrated affinity, promoted potential uptake of vegetables from soil, and a common source of pollution. Hence, expanding the current research to include determining which part of a vegetable stores Ni, contaminant transport analysis, biomonitoring of Cr and Ni, different routes of metal exposure such as eating other foods (root crops, fruits, and cereals), drinking water, and air contact, uncertainty and variability analyses, is crucial to further the understanding of the risks associated with the elevated metal concentrations in the soil and its produce on human health, especially targeting carcinogenic risk. Further, the result of this study is useful for the development of more focused studies for more specific contaminants and edible agricultural yields that are consumed by the public. In addition, epidemiological studies specific to cancer risks are useful to help health authorities and relevant government agencies to create necessary programs to protect public health.

## Figures and Tables

**Table 1:** The grading standards for pollution indices [44].

Class of Pollution	I	II	III	IV	V
$P_{i}$	≤1.0	1.0–2.0	2.0–3.0	3.0–5.0	>5.0
$P_{n}$	≤0.7	0.7–1.0	1.0–2.0	2.0–3.0	>3.0
Pollution level	Clean	Warning	Light	Intermediate	Severe
Risk index	<150	150–300	300–600	600–1200	>1200
Pollution risk	Low	Moderate	Considerable	High	Very high
	(3)				

**Table 2:** Mean concentrations of metals in soil (${\mathrm{mg}\;\mathrm{kg}}^{-1}$) with soil quality standards (SQS), *n* = 90.

Location	Metal							
	Cd	Cr	Cu	Fe	Mn	Ni	Pb	Zn
Boac	0	854 ±365	9159 ± 10,727	772,972 ± 116,476	25,597 ± 20,945	567 ± 285	664 ± 333	3734 ± 2083
Buenavista	0	1695 ± 921	2090 ± 827	759,560 ± 325,617	60,550 ± 52,526	1016 ± 954	930 ± 636	3734 ± 2083
Gasan	0	2466 ± 1380	1711 ± 358	1,083,607 ± 616,615	29,238 ± 9882	3216 ± 3412	393 ± 108	2291 ± 494
Mogpog	0	862 ± 143	17,712 ± 15,394	783,457 ± 133,191	31,893 ± 9421	536 ± 109	1291 ± 431	6161 ± 2962
Sta. Cruz	0	1257 ± 917	3506 ± 857	1,030,753 ± 197,825	50,522 ± 38,289	898 ± 830	645 ± 334	2760 ± 1014
Torrijos	0	1409 ± 533	2807 ± 1328	762,505 ± 188,812	38,298 ± 15,042	722 ± 463	682 ± 203	2790 ± 876
SQS	0.15 ^1^	80 ^1^	45 ^1^	NA	100 ^2^	45 ^1^	55 ^1^	70 ^1^

^1^ Chen et al., 2018, ^2^ Semenkov et al., 2020.

**Table 3:** Trend of metal concentrations in the soil across the province.

Location	Metals in Soil
Boac	Fe > Mn > Zn > Cu > Cr > Ni > Pb > Cd
Buenavista	Fe > Mn > Zn > Cu > Cr > Pb > Ni > Cd
Gasan	Fe > Mn > Cu > Zn > Cr > Ni > Pb > Cd
Mogpog	Fe > Mn > Zn > Cu > Cr > Ni > Pb > Cd
Sta. Cruz	Fe > Mn > Cu > Zn > Cr > Ni > Pb > Cd
Torrijos	Fe > Mn > Cu > Zn > Ni > Cr > Pb > Cd

**Table 4:** Distribution trend of metal concentration in vegetables.

Location	Vegetable			
	String Beans	Sweet Potato Tops	Bitter Melon	Eggplant
Boac	Zn > Fe > Mn > Cu > Pb > Cr > Ni > Cd	Fe > Zn > Mn > Ni > Cu > Pb > Cd > Cr	Zn > Fe > Cu > Mn > Ni > Pb > Cd > Cr	Zn > Fe > Cu > Mn > Ni > Pb > Cd > Cr
Buenavista	Zn > Fe > Mn > Cu > Pb > Cr > Ni > Cd	Fe > Zn > Mn > Ni > Cu > Pb > Cd > Cr	Zn > Fe > Mn > Ni > Cu > Pb > Cd > Cr	Cu > Mn > Ni > Fe > Pb > Cd > Zn > Cr
Gasan	Zn > Fe > Mn > Cu > Pb > Cr > Ni > Cd	Fe > Zn > Mn > Ni > Cu > Cr > Pb > Cd	Zn > Fe > Mn > Ni > Cu > Cr > Pb > Cd	Zn > Fe > Mn > Ni > Cu > Cr > Pb > Cd
Mogpog	Zn > Fe > Mn > Ni > Cu > Cr > Pb > Cd	Fe > Zn > Mn > Ni > Cu > Cr > Pb > Cd	Zn > Fe > Mn > Ni > Cu > Cr > Pb > Cd	Zn > Fe > Mn > Ni > Cu > Cr > Pb > Cd
Sta. Cruz	Zn > Fe > Mn > Ni > Cu > Cr > Pb > Cd	Zn > Fe > Mn > Ni > Cu > Cr > Pb > Cd	Zn > Fe > Mn > Ni > Cu > Cr > Pb > Cd	Zn > Fe > Mn > Ni > Cu > Cr > Pb > Cd
Torrijos	Zn > Ni > Fe > Mn > Cr > Pb > Cu > Cd	Fe > Zn > Mn > Ni > Cu > Cr > Pb > Cd	Zn > Mn > Ni > Fe > Cu > Cr > Pb > Cd	Zn > Fe > Mn > Ni > Cr > Cu > Pb > Cd

**Table 5:** The estimated daily intake (EDI) of metals ${\mathrm{mg}\;\mathrm{kg}}^{-1}\;{\mathrm{day}}^{-1}$ through vegetables.

Location	Vegetable	Cd	Cr	Cu	Fe	Mn	Zn	Ni	Pb
Boac	String beans	3.85 × 10^−8^	5.67 × 10^−9^	1.92 × 10^−4^	5.10 × 10^−4^	2.82 × 10^−4^	1.73 × 10^−3^	6.00 × 10^−9^	1.28 × 10^−7^
	Sweet potato tops	4.29 × 10^−8^	6.31 × 10^−9^	2.39 × 10^−4^	1.31 × 10^−3^	3.31 × 10^−4^	1.26 × 10^−3^	6.68 × 10^−9^	1.43 × 10^−7^
	Bitter melon	4.09 × 10^−8^	6.02 × 10^−9^	1.48 × 10^−4^	4.67 × 10^−4^	1.71 × 10^−4^	1.85 × 10^−3^	6.37 × 10^−9^	1.36 × 10^−7^
	Eggplant	7.71 × 10^−8^	1.13 × 10^−8^	2.20 × 10^−4^	6.76 × 10^−5^	1.53 × 10^−4^	1.85 × 10^−8^	1.20 × 10^−8^	2.57 × 10^−7^
Buena-vista	String beans	4.83 × 10^−5^	7.34 × 10^−5^	1.20 × 10^−4^	6.95 × 10^−4^	3.32 × 10^−4^	2.01 × 10^−3^	1.21 × 10^−4^	6.31 × 10^−5^
	Sweet potato tops	5.27 × 10^−5^	8.44 × 10^−5^	1.56 × 10^−4^	4.12 × 10^−3^	6.20 × 10^−4^	1.45 × 10^−3^	6.42 × 10^−5^	6.55 × 10^−5^
	Bitter melon	5.05 × 10^−5^	7.72 × 10^−5^	1.83 × 10^−4^	4.80 × 10^−4^	1.84 × 10^−4^	2.10 × 10^−3^	6.25 × 10^−5^	6.22 × 10^−5^
	Eggplant	9.56 × 10^−5^	1.44 × 10^−4^	2.64 × 10^−4^	6.42 × 10^−4^	4.27 × 10^−4^	1.65 × 10^−3^	1.09 × 10^−4^	1.17 × 10^−4^
Gasan	String beans	4.77 × 10^−5^	7.33 × 10^−5^	1.55 × 10^−4^	7.04 × 10^−4^	3.77 × 10^−4^	2.31 × 10^−3^	1.47 × 10^−4^	6.07 × 10^−5^
	Sweet potato tops	5.31 × 10^−5^	1.09 × 10^−4^	2.00 × 10^−4^	7.31 × 10^−3^	5.03 × 10^−4^	1.85 × 10^−3^	1.29 × 10^−4^	7.72 × 10^−5^
	Bitter melon	5.08 × 10^−5^	7.79 × 10^−5^	1.11 × 10^−4^	5.34 × 10^−4^	4.20 × 10^−4^	1.69 × 10^−3^	8.74 × 10^−5^	6.42 × 10^−5^
	Eggplant	9.86 × 10^−5^	1.46 × 10^−4^	3.14 × 10^−4^	8.35 × 10^−4^	4.10 × 10^−4^	3.16 × 10^−3^	1.37 × 10^−4^	1.38 × 10^−4^
Mogpog	String beans	1.32 × 10^−6^	5.67 × 10^−9^	1.49 × 10^−4^	6.48 × 10^−4^	1.76 × 10^−4^	1.64 × 10^−3^	6.00 × 10^−9^	1.28 × 10^−7^
	Sweet potato tops	4.29 × 10^−8^	6.31 × 10^−9^	2.93 × 10^−4^	3.60 × 10^−3^	3.44 × 10^−4^	1.55 × 10^−3^	6.68 × 10^−9^	1.43 × 10^−7^
	Bitter melon	4.09 × 10^−8^	6.02 × 10^−9^	1.84 × 10^−4^	4.22 × 10^−4^	1.31 × 10^−4^	2.13 × 10^−3^	6.37 × 10^−9^	1.36 × 10^−7^
	Eggplant	7.71 × 10^−8^	1.13 × 10^−8^	3.76 × 10^−4^	4.12 × 10^−4^	3.92 × 10^−5^	2.32 × 10^−3^	1.20 × 10^−8^	2.57 × 10^−7^
Sta. Cruz	String beans	3.85 × 10^−8^	5.92 × 10^−6^	1.81 × 10^−4^	5.77 × 10^−4^	2.40 × 10^−4^	2.90 × 10^−3^	9.90 × 10^−6^	1.23 × 10^−5^
	Sweet potato tops	4.29 × 10^−8^	9.57 × 10^−6^	1.46 × 10^−4^	8.31 × 10^−4^	3.37 × 10^−4^	1.85 × 10^−3^	3.53 × 10^−6^	2.73 × 10^−5^
	Bitter melon	4.09 × 10^−8^	6.04 × 10^−6^	8.89 × 10^−5^	4.51 × 10^−4^	1.26 × 10^−4^	1.55 × 10^−3^	1.49 × 10^−5^	2.76 × 10^−6^
	Eggplant	7.71 × 10^−8^	1.79 × 10^−5^	3.20 × 10^−4^	5.25 × 10^−4^	4.21 × 10^−4^	1.93 × 10^−3^	4.59 × 10^−5^	3.29 × 10^−6^
Torrijos	String beans	3.85 × 10^−8^	8.88 × 10^−6^	1.35 × 10^−4^	7.22 × 10^−4^	3.67 × 10^−4^	2.36 × 10^−3^	1.08 × 10^−5^	2.88 × 10^−6^
	Sweet potato tops	4.29 × 10^−8^	9.51 × 10^−6^	1.30 × 10^−4^	1.64 × 10^−3^	4.66 × 10^−4^	1.80 × 10^−3^	5.25 × 10^−6^	3.42 × 10^−6^
	Bitter melon	4.09 × 10^−8^	8.17 × 10^−6^	9.69 × 10^−5^	5.26 × 10^−4^	1.88 × 10^−4^	1.66 × 10^−3^	6.60 × 10^−6^	4.07 × 10^−6^
	Eggplant		1.55 × 10^−5^	1.89 × 10^−4^	3.86 × 10^−4^	2.68 × 10^−4^	1.76 × 10^−3^	3.76 × 10^−6^	8.24 × 10^−6^

**Table 6:** Target cancer risk due to the consumption of vegetables with elevated heavy metal concentrations.

Location	Cr	Cd	Ni	Pb
Boac	1.47 × 10^−8^	7.57 × 10^−8^	5.28 × 10^−8^	5.65 × 10^−9^
Buenavista	1.89 × 10^−4^_1_	9.39 × 10^−5^	6.08 × 10^−4^_1_	2.62 × 10^−6^
Gasan	2.03 × 10^−4^_1_	9.51 × 10^−5^	8.51 × 10^−4^_1_	2.89 × 10^−6^
Mogpog	1.47 × 10^−8^	5.62 × 10^−7^	5.28 × 10^−8^	5.65 × 10^−9^
Sta. Cruz	1.97 × 10^−5^	7.57 × 10^−8^	1.26 × 10^−4^_1_	3.87 × 10^−7^
Torrijos	2.10 × 10^−5^	6.20 × 10^−8^	4.49 × 10^−5^	1.58 × 10^−7^

_1_ Exceeded the maximum threshold value ($1.00\times 10^{-4}$) for the risk of developing cancer.

**Table 7:** The target cancer risk at 75% reduction of vegetable consumption.

Location	Cr	Cd	Ni	Pb
Boac	2.96 × 10^−9^	1.53 × 10^−8^	1.06 × 10^−8^	1.14 × 10^−9^
Buenavista	4.73 × 10^−5^_1_	2.35 × 10^−5^	1.52 × 10^−4^_1_	6.55 × 10^−7^
Gasan	5.08 × 10^−5^_1_	2.38 × 10^−5^	2.13 × 10^−4^_1_	7.22 × 10^−7^
Mogpog	8.80 × 10^−9^	3.37 × 10^−7^	3.17 × 10^−8^	3.39 × 10^−9^
Sta. Cruz	4.93 × 10^−6^	1.89 × 10^−8^	3.15 × 10^−5^_1_	9.68 × 10^−8^
Torrijos	5.26 × 10^−6^	1.55 × 10^−8^	1.12 × 10^−5^	3.96 × 10^−8^

_1_ Exceeded the maximum threshold value ($1.00\times 10^{-4}$) for the risk of developing cancer.

**Table 8:** Correlation matrix of metals in the soil.

Metals	Cr	Fe	Mn	Ni	Pb	Zn	Cu
Cr	1	0.600	0.131	0.917 **	−0.602	−0.640	−0.708
Fe		1	−0.060	0.746 *	−0.624	−0.555	−0.395
Mn			1	−0.194	0.165	−0.142	−0.457
Ni				1	−0.627	−0.535	−0.478
Pb					1	0.940 **	0.761 *
Zn							0.921 **
Cu							1

** Correlation is significant at the 0.01 level (2-tailed). * Correlation is significant at the 0.05 level (1-tailed).

**Table 9:** Correlation matrix of metals in the vegetables.

Metals	Cd	Cr	Fe	Mn	Ni	Pb	Zn	Cu
Cd	1	0.986 **	0.303	0.439 *	0.387	0.977 **	0.082	−0.139
Cr		1	0.409 *	0.502 *	0.462 *	0.977 **	0.104	−0.150
Fe			1	0.629 **	0.605 **	0.325	0.014	0.311
Mn				1	0.852 **	0.443 *	0.274	0.031
Ni					1	0.399	0.168	0.002
Pb						1	0.077	−0.106
Zn							1	0.113
Cu								1

** Correlation is significant at the 0.01 level (2-tailed). * Correlation is significant at the 0.05 level (2-tailed).

**Table 10:** The spatial autocorrelation and its interpretation.

Metals	Media	Moran’s I	Z-Score	*p*-Value	Remarks
Cd	Soil	-	-	-	-
	Vegetables ^1^	0.668301	2.857647	0.004269	There is <1% likelihood that this clustered pattern could be the result of random chance.
Cr	Soil ^1^	0.600711	3.66462	0.000248	There is <1% likelihood that this clustered pattern could be the result of random chance.
	Vegetables ^1^	0.700762	3.002888	0.002674	There is <1% likelihood that this clustered pattern could be the result of random chance.
Cu	Soil ^2^	0.157930	1.323145	0.185787	The pattern does not appear to be significantly different than random.
	Vegetables ^2^	0.313179	1.500966	0.133364	The pattern does not appear to be significantly different than random.
Fe	Soil ^2^	−0.078156	−0.290135	0.771713	The pattern does not appear to be significantly different than random.
	Vegetables ^2^	−0.063573	−0.101530	0.919130	The pattern does not appear to be significantly different than random.
Mn	Soil ^2^	0.010371	0.284159	0.776288	The pattern does not appear to be significantly different than random.
	Vegetables ^1^	0.411650	1.878665	0.06029	There is <10% likelihood that this clustered pattern could be the result of random chance.
Ni	Soil ^1^	0.517934	4.732331	0.000002	There is <1% likelihood that this clustered pattern could be the result of random chance.
	Vegetables ^1^	0.591938	2.662094	0.007766	There is <1% likelihood that this clustered pattern could be the result of random chance.
Pb	Soil ^1^	0.300678	1.957617	0.050275	There is <10% likelihood that this clustered pattern could be the result of random chance.
	Vegetables ^1^	0.742895	3.158990	0.001583	There is <1% likelihood that this clustered pattern could be the result of random chance.
Zn	Soil ^1^	0.339623	2.150663	0.031503	There is <5% likelihood that this clustered pattern could be the result of random chance
	Vegetables ^2^	−0.210062	−0.720262	0.471363	The pattern does not appear to be significantly different than random.

^1^ Reject null hypothesis, dataset is more spatially clustered than would be expected. ^2^ Spatial distribution of feature values is the result of random processes.

**Figure A1:**
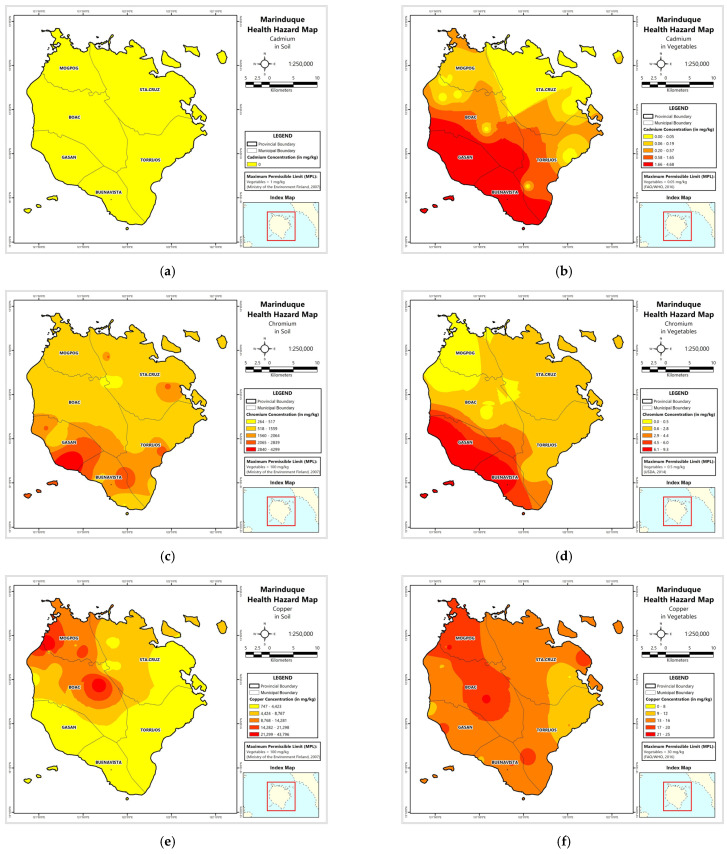

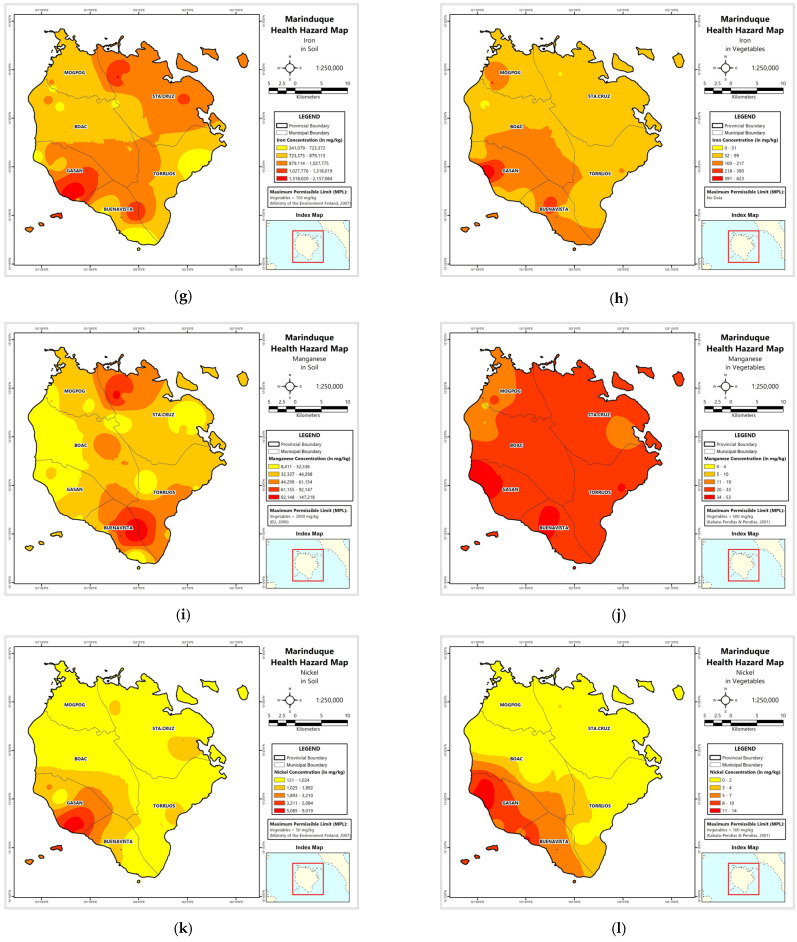

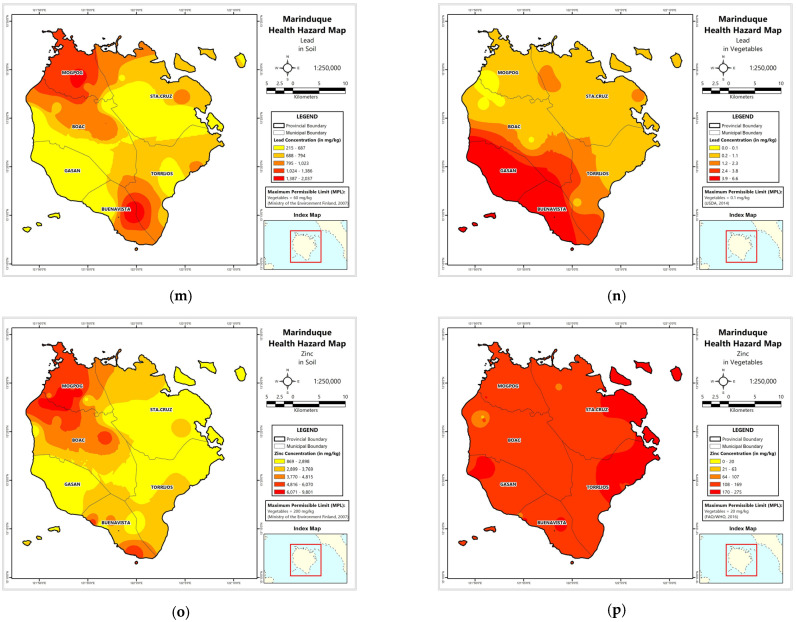
Spatial distribution maps of metal concentrations in soil and vegetables. (**a**) Soil–Cd; (**b**) Vegetables–Cd; (**c**) Soil–Cr; (**d**) Vegetables–Cd; (**e**) Soil–Cu; (**f**) Vegetables–Cu; (**g**) Soil–Fe; (**h**) Vegetables–Fe; (**i**) Soil–Mn; (**j**) Vegetables–Mn; (**k**) Soil–Ni; (**l**) Vegetables–Ni; (**m**) Soil–Pb; (**n**) Vegetables–Pb; (**o**) Soil–Zn; (**p**) Vegetables–Zn.

## Data Availability

All data are contained in the manuscript.
